# Horizontal Spatial Metaphor Representation of Social Status in Chinese Culture

**DOI:** 10.3389/fpsyg.2021.658161

**Published:** 2021-11-29

**Authors:** Hongri Sun, Danfeng Liu

**Affiliations:** School of Public Administration, Nanchang University, Nanchang, China

**Keywords:** social status, horizontal spatial metaphor, embodiment effect, implicit relational assessment procedure, spatial placement task

## Abstract

The mapping relationship between social status and horizontal space (left/right) in Chinese culture has a long history. In order to explore the representation pattern of horizontal spatial metaphor of social status in Chinese culture, this study introduced two direct measurements, implicit relational assessment procedure (IRAP) paradigm, and spatial placement task to evaluate the mapping of social status to horizontal space. A total of 144 Chinese undergraduates participated in the research, wherein they were asked to place certain words indicating social status in either left or right box before or after the IRAP computer test. The results from the two measurements consistently showed that the mode of HIGH SOCIAL STATUS-LEFT and LOW SOCIAL STATUS-RIGHT (HLLR) had an advantage over HIGH SOCIAL STATUS-RIGHT and LOW SOCIAL STATUS-LEFT (HRLL), implying that the representation pattern of horizontal spatial metaphor of social status for the Chinese is HIGH SOCIAL STATUS-LEFT and LOW SOCIAL STATUS-RIGHT. However, the result convergence of the two measurements was not high, which suggests that embodiment effect has multiple characteristics and new specific experimental paradigms should be created to measure it.

## Introduction

Conceptual metaphors are one of the cognitive tools used for abstract thinking, through which complex and abstract concepts can be understood and represented using simpler and more concrete concepts (Lakoff and Johnson, 1980; Wilson, 2002). For example, the expression “Social status is high/low” uses the tangible experiences of the perception over the vertical axis (high/low) to help us metaphorically perceive and understand the concept of social status, which is otherwise abstract and complex. According to the Conceptual Metaphor Theory (CMT), it is “our physical and cultural experience” that provides the basis for conceptual metaphors (Lakoff and Johnson, 1980).

As a complex and abstract concept, social status is often perceived and processed using spatial information (Gagnon et al., 2011). Studies have shown that response time was faster when the labels of high social status were located visually at the upper side of the screen when compared to the lower side of the screen, and vice versa (Schubert, 2005). The classifying speed was faster when the images of influential people/mountains and ordinary people/plains shared the same reaction button than when they shared different reaction buttons in Implicit Classification task, using figures and images of influential people/ordinary people and topographic images of mountains/plains (Gagnon et al., 2011, Experiment 1). When directly evaluating the relationship between high/low social status and upper/lower vertical space, the response latencies were shorter in the compatible task (high social status located at the upper part of the screen and low social status at the lower part of the screen were judged as consistent and as inconsistent if otherwise) when compared to the incompatible task (high status located at the lower part of the screen and low status at the upper part of screen were judged as consistent, and as inconsistent if otherwise) (Hong et al., 2019). By constructing hierarchical representations of social status among multiple virtual target characters, Hecker et al. (2013) further confirmed that social status was metaphorically mapped onto vertical space. Moreover, this spatial representation mode was independent of social status distance. It can be infer through hierarchical order transmission for the external pair even if they were nonadjacent social status ranks. This suggests that the vertical spatial location of a person conveys meaningful information about his/her social status (Lamer et al., 2020).

Compared with the vertical spatial metaphor, the horizontal spatial metaphor of social status is less influential. However, in traditional Chinese ranking culture, social status is more interrelated to “noble” (high social status) and “humble” (low social status). Since ancient times, the concept of social status has gradually been linked with natural orientation. For example, high social status families are referred to as the Right Clan while the common people are called Lu Left (“Lu” is the gate of an alley) in History books. In the daily life of the Chinese, the social status of a person determines their allocated side (i.e., left or right), when they need to be arranged spatially over a horizontal axis (Sun, 2010). For example, the elders are always seated on the left while deciding seating arrangements, as a mark of respect. However, the allocated pattern is complex and diverse, which sometimes maps high social status to the left and at other times to the right (Qian, 2013). Life experiences may play a symbolic role that directly relates to perception and influences our mental representations implicitly and explicitly (Gagnon et al., 2011). However, agency and power, two concepts that are closely similar to social status in describing personal relationships, are proven to present specific patterns of horizontal spatial metaphorical representations (Maass et al., 2009; Mendona et al., 2020). Maass et al. (2014) verified that the pattern is more related to the language structure through cross-cultural research. The direction of writing/reading and the subject–object relations that are mentioned both contribute to the horizontal spatial metaphor of concepts. Agency is linked to the left and recipients to the right in languages with script from left to right, and objects are mentioned after subject words. Currently, the mainstream direction of writing/reading is from the left to right, and the word order is usually subject–verb–object in the Chinese language. If the findings of Maass et al. (2014) were to generalize the social status, it could be inferred that the representational pattern of the horizontal spatial metaphor of social status should be HIGH SOCIAL STATUS-LEFT and LOW SOCIAL STATUS-RIGHT; however, this is not completely consistent with the pattern in the daily life of the Chinese. Therefore, it is interesting to evaluate the pattern of social status within the Chinese culture.

In nature, the representation of conceptual metaphors is a construct with unobservable properties. Methodologically, evaluating it using various measurements is a good practice to capture the construct more comprehensively and accurately. Implicit Relational Assessment Procedure (IRAP) paradigm and the spatial placement task were introduced to measure the constructs in this study. The IRAP is an automated computer task that directly measures the relationship between stimuli. In light of the Relational Framework Theory (RFT) (Barnes–Holmes et al., 2001), cognitive function is composed of relational behavior, and the core of understanding human language is through relationships (Barnes–Holmes et al., 2006). When exposed to a given stimuli relationship (the relationship of mapping social status on horizontal space) without the pressure of time and cognition, participants can produce a response coherent with one or more relational responses, such as “the one with high social status should be on the left,” and “At present, it is a society where everyone is equal, so we should not treat people differently” in the task of the explicit measurement of spatial metaphors of social status, such as the spatial placement task. Under this condition, a more carefully considered relational response is measured. However, there is “insufficient time for such elaborated relational responding in a time-pressured IRAP,” and it just captures spontaneous and automatic impressions driven largely by immediate and relatively brief relational responses (Barnes–Holmes et al., 2010). Theoretically, the results of both the measurements should be the same or highly correlated because they evaluate the same concept or construct. However, Lamer et al. (2020) found that the result of the spatial placement task could not be repeated by the spatial memory task for social group identity. Participants were asked to directly place objects symbolizing femininity and masculinity on a blank refrigerator, for the spatial placement task, and to recall the position where the specific magnets were located in the refrigerator, for the spatial memory task. The results from the two methods were inconsistent. In this study, the spatial memory task was replaced by IRAP because the evaluating process of IRAP was more similar to the spatial placement task in which the participants directly reacted to mapping relationships. The purpose was to answer what the horizontal spatial metaphor representation of social status is, in Chinese culture, and to assess the convergence between the two measurements.

## Methods

### Participants

A total of 166 Chinese undergraduates participated in the research. Data from 144 participants (age ranging from 17 to 25, *M* = 19.78, *SD* = 1.59; 87 women) were collected, excluding invalid data from 22 participants. The *post hoc* of power (1-β err prob) with 144 participants was 0.49 based on the G-power (Faul et al., 2007) for the correlation *t*-test at the effect size | ρ| = 0.16. The participants were all right-handed, did not have dyslexia, were familiar with computer operations, and their mother tongue was Chinese. All participants signed the written informed consent.

### Stimuli

Twelve words referring to the experimental materials of Schubert (2005) and Hong et al. (2019) were selected, including six high social status (HSS) words [general (将军), emperor (皇帝), academician (院士), professor (教授), president (总裁), and judge (法官)] and six low social status (LSS) words [security guard (保安), worker (工人), prisoner (囚犯), coachman (车夫), slave (奴隶), and servant (仆人)]. Before the experiment, 45 Chinese undergraduates (25 women) who did not participate in the experiment evaluated the 12 words, along with the dimensions of social status rank and familiarity, from 1 (lowest/unfamiliar) to 5 (highest/familiar). A comparison of the matched pairs showed that the social status ranking was significantly different between HSS and LSS [*M*_HSS_ = 4.43, *SD*_HSS_ = 0.45; *M*_LSS_ = 1.71, *SD*_LSS_ = 0.65; *t*_(44)_ = 20.27, *p* < 0.001, Cohen *d* = 3.02, 95% CI (2.46, 2.96)], while familiarity was not [*M*_HSS_ = 3.90, *SD*_HSS_ = 0.92; *M*_LSS_ = 3.88, *SD*_LSS_ = 0.96; *t*_(44)_ = 0.16, *p* = 0.873, Cohen *d* = 0.02, 95% CI (−0.17, 0.21)]. Compared with 3 (general familiarity) using one-sample *t*-test, the group level of the words’ familiarity were both significantly over 3 [*t*_(44)HSS_ = 6.55, *p*_HSS_ < 0.001, Cohen *d*_HSS_ = 1.38, 95% CI_HSS_ (0.65,1.14); *t*_(44)LSS_ = 6.17, *p*_LSS_ < 0.001, Cohen *d*_LSS_ = 1.30, 95% CI_LSS_ (0.62,1.16)].

### Measurements

#### IRAP Paradigm

The IRAP paradigm adopted the classic eight-stage procedure (Barnes–Holmes et al., 2006) and was run in E-prime 2.0.

As shown in Table 1, in each trial, one of the 12 words (printed in Song typeface font, size 32) was chosen and displayed randomly on the center of the right or left half of the screen. Participants were asked to judge whether the relationship between the word and its displayed orientation (based on the left- and right-hand of the participants) was consistent according to the guidance of introduction, and subsequently press the key on the corresponding side of the answer to make a selection. The left and right positions of “consistent” and “inconsistent” were random. If the answer was on the left side, the participants pressed the “D” key to make a selection and enter into the next trial, otherwise the “K” key was pressed. If no key was pressed within 10,000 ms, the next trial would automatically begin and the answer of the previous trial would be marked as wrong. Participants were reminded that it was better to respond quickly and accurately. Each word was displayed once on each side at every stage. Thus, there were 24 trials altogether in each stage.

**TABLE 1:** The construct of IRAP procedure.

Stage	Stimuli Relationship	Response		IRAP effect	Phase
		Version 1	Version 2		
1	HL/LR	Consistent	Inconsistent		training
	HR/LL	Inconsistent	Consistent		
2	HL/LR	Consistent	Inconsistent		
	HR/LL	Inconsistent	Consistent		
3	HL/LR	Consistent	Inconsistent		
	HR/LL	Inconsistent	Consistent		
4	HL/LR	Consistent	Inconsistent		
	HR/LL	Inconsistent	Consistent		
5	HL/LR	Consistent	Inconsistent	D_IRAP1_	testing
	HR/LL	Inconsistent	Consistent		
6	HL/LR	Consistent	Inconsistent		
	HR/LL	Inconsistent	Consistent		
7	HL/LR	Consistent	Inconsistent	D_IRAP2_	
	HR/LL	Inconsistent	Consistent		
8	HL/LR	Consistent	Inconsistent		
	HR/LL	Inconsistent	Consistent		

*HL, LR, HR, and LL are four types of stimuli relationships. The first letter represents the rank of social status, high or low; the last letter represents the displayed position of social status words, in the center of right or left half of screen. For example, HL = high social status word was displayed in the center of left half of screen.*

There were four types of stimuli relationships between the high/low social status and left/right spatial positions—HIGH SOCIAL STATUS-LEFT (HL), HIGH SOCIAL STATUS-RIGHT (HR), LOW SOCIAL STATUS-LEFT (LL), and LOW SOCIAL STATUS-RIGHT (LR). They could form two distinct patterns—HIGH SOCIAL STATUS-LEFT and LOW SOCIAL STATUS-RIGHT (HLLR); and HIGH SOCIAL STATUS-RIGHT and LOW SOCIAL STATUS-LEFT (HRLL)—which clearly distinguish the difference in the orientation of the horizontal spatial mapping of high/low social status. Therefore, they were used as two different tasks in IRAP, and each stage presented one of them. For all the eight stages in IRAP, the two tasks (i.e., patterns) were presented in turn. The first four stages were the training phase in which a red “×” followed wrong responses, and the participants were required to correct the mistakes before entering the next trial. The last four stages were the testing phase in which all the 24 trials of each stage were presented one by one without any mistake in feedback and correction. Two different IRAP versions beginning with the HLLR or the HRLL tasks were counterbalanced among the participants.

The difference in the IRAP effect between the two IRAP versions was not significant [*M*_HLLR_ = 0.01, *SD*_HLLR_ = 0.27; *M*_HRLL_ = 0.09, *SD*_HRLL_ = 0.24; *t*_(83,59)_ = −1.82, *p* = 0.070, Cohen *d* = 0.16, 95% CI (−0.17, 0.01)].

#### Spatial Placement Task

**FIGURE 1:**
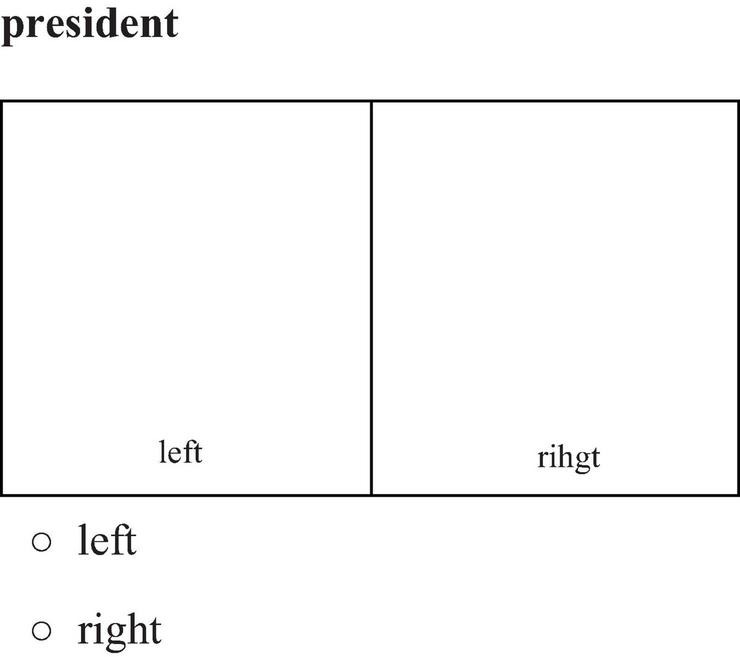
Spatial placement task (the upper and lower space positions of the two options were random).

The participants were asked to judge and choose the side (right/left) of the rectangle on which the social status words should be displayed, without time pressure. In each trial, a horizontal rectangular box (386 × 215 pixels) was divided into two equal halves by a vertical line that was displayed in the center of the screen. The terms “left” and “right” were marked at the bottom center of each half, respectively, referring to the left- and right-hand horizontal spatial orientations of the participants. The social status word appeared at the upper left side above the rectangular box, and the “left” and “right” option buttons at the lower left side under the rectangular box (as shown in Figure 1). The upper and lower spatial positions of the two options were random. The participants were asked to decide the side of the rectangle (right/left based on the left- and right-hand of the participants) on which they wanted to put the word and click the corresponding option using the left button of a computer mouse. In other words, if they chose to place the word on the left, the “left” option under the rectangle should be clicked, conversely, “right” option should be clicked. Participants were allowed to change their choices without limitation, before pressing the OK button for the next trial.

Only one word was displayed in each trial. The first three trials were used for practicing how to choose and press the options. Words were randomly selected from the 12 social status words. This was followed by 12 trials for the 12 words, which were displayed randomly without repetition. Therefore, there were 15 trials altogether.

### Procedure

All participants completed the two measurements successively. After signing the written informed consent, the participants were randomly divided into two groups, and each group was assigned to one of the IRAP versions (the procedure beginning with the HLLR or the HRLL task). Each group was further divided randomly into two subgroups, where one performed the IRAP first while the other performed the space placement task first. After measurement, the participants were asked to complete the personal information questionnaire and were paid RMB 5 as a reward.

### Data Processing

The data of 22 participants were excluded owing to a lower than 80% accuracy rate in any IRAP stage during the testing phase. According to the D algorithm (Greenwald et al., 2003), eight trials (about 0.058%) with reaction times (RT) below 300 ms were recorded as 300 ms, along with 719 trials (about 5.2%) above 3,000 ms, which were recorded as 3,000 ms (total number of trials were 13,824). Four *D-*values were then obtained by dividing the mean RT difference between HLLR and HRLL in stages 5 and 6 of the IRAP by pooling the standard deviations of the four types of stimulus combinations: HL, HR, LL, and LR; and D_IRAP1_ was produced by averaging all of the four *D-*values. The same procedure was followed for the stages 7 and 8 of the IRAP and in producing D_IRAP2_. The total IRAP effect of the horizontal spatial metaphors of social status, obtained by averaging the sum of D_IRAP1_ and D_IRAP2_, was denoted by D_IRAP_.

In the spatial placement task, it was coded 1 when the high social status word was located on the left side or the low social status word was located on the right side; otherwise, it was coded 0. Thus, the sum of all the 12 words was the score of spatial placement task, and it ranged from 0 to 12. Two scores, 12 and 0, could be clearly identified with HLLR and HRLL, respectively, because they indicated that all the words representing the same social status rank were located on the same side and that different ranks were on opposite sides. The rest were marked as “uncertain” (UN) because the location of the words indicated by the scores was indeterminate.

## Results

### Spatial Metaphorical Representation of Social Status

A one-sample *t*-test was used with a 95% confidence interval (CI) with Bootstrap self-sampling done 1,000 times to investigate whether there was an IRAP effect on the mapping relationship between social status and horizontal space. The D_IRAP_ was greater than 0 [*M* = 0.05, *SD* = 0.26, *t*_(143)_ = 2.10, *p* = 0.038, Cohen’s *d* = 0.25, 95% CI (0.004, 0.092)], implying that the response time for the HLLR mode was faster than for the HRLL mode. However, the effect size was small.

**FIGURE 2:**
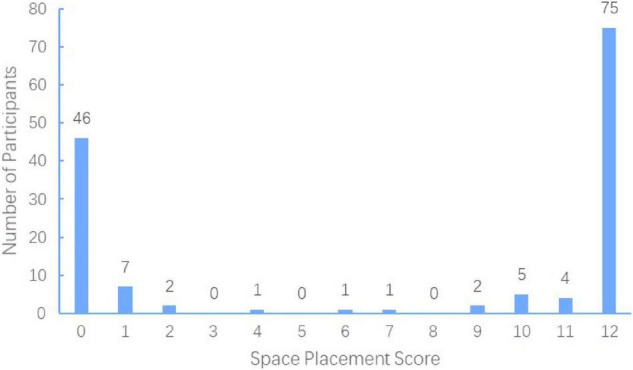
Number of participants for different scores in spatial placement task. Score 12 and 0 could be identified as mode HLLR (HIGH SOCIAL STATUS-LEFT and LOW SOCIAL STATUS-RIGHT) and HRLL (HIGH SOCIAL STATUS-RIGHT and LOW SOCIAL STATUS-LEFT), respectively; Scores from 1 to 11 could not be identified as a given mode, so they were classified as mode UN (UNCERTAIN).

For the spatial placement task, 75 (52%) participants scored 12, while 46 (32%) scored 0, and 23 (16%) did not score 0 or 12 (see Figure 2). The χ^2^ test showed that the number of participants divided into different categories according to the scores were significantly different [χ^2^_(2)_ = 28.29, *p* < 0.001, *w* = 0.44], and more participants scored 12 rather than 0 [χ^2^_(1)_ = 6.95, *p* = 0.008 < 0.05/3, w = 0.24]. The results showed that more participants placed the words of high social status in the left-hand space area, while words of low social status were placed in the right-hand space area. This meant that most of the representations of the spatial metaphors of social status by participants were HLLR.

### Correlation Between IRAP Effect and Space Placement Score

The Spearman’s correlation coefficient for ranked data was used to investigate the relationship between D_IRAP_ and the space placement score because of the non-normal distribution of the latter variable (Skewness = −0.43, Kurtosis = −1.79, *W* = 0.67, *p* < 0.001). The result showed that the correlation of the two variables was not significant (ρ = 0.16, *p* = 0.063), which implied that the correlation between the IRAP effect and the spatial placement score was not high.

## Discussion

### Horizontal Spatial Metaphor Representation of Social Status in Chinese Culture

When we arrange people of different social statuses in a horizontal space, we do not do it randomly; they are located in special patterns. This study used the implicit measurement IRAP paradigm and the spatial placement task to explore the horizontal spatial metaphor representation of social status in Chinese culture. The results showed that the D_IRAP_ value was above 0, implying that the participants responded faster for the HLLR mode rather than for HRLL. Meanwhile, most of the participants (52%) placed high social status group words on the left-hand side and low social status group words on the right-hand side, thereby proving that the metaphorical representation of social status in Chinese culture is in the HLLR mode.

In 2003, Maass and Russo found that the influence of writing habits on horizontal spatial metaphors was far greater than that of hemispheric specialization; Italians tended to place subject words to the left of the object words, while Arabic tended to place the subject words to the right (Maass and Russo, 2003). Further studies found that Italian users tend to put the active group (men and young people) on the left of the passive group (women and the elderly), while Arabic users tend to put the active groups on the right. Therefore, the researchers proposed the model of spatial agency bias (SAB), which suggested that the horizontal spatial representation of social groups is related to the writing/reading direction of the mainstream culture (Maass et al., 2009). In Malagasy participants, Maass et al. (2014) further found that the writing/reading direction was not enough to explain the horizontal spatial representation of agency and that subject–object order was also an important factor, which extended SAB to form the model of dual process of SAB. Studies also found that this kind of spatial bias may have been unconsciously transmitted to the next generation in the cultural group through spontaneous gestures or activities of caregivers during parent–child activities, before children form the habit of automatic writing (McCrink et al., 2018). The native language of the participants in this study was Chinese, which has the same writing/reading directions and sentence order as Italian. According to the SAB model (Maass et al., 2014), this may explain why the HLLR mode has an advantage over that of the HRLL mode in the horizontal metaphorical representation of social status in the Chinese culture. Just like the concept of agency, the SAB can predict the horizontal spatial metaphorical patterns of the concept of social status, at least for the Chinese speaker.

### Convergence of Two Measurements for Horizontal Spatial Metaphor of Social Status

The approach of evaluating the same concept or construct through two measurements is based on their high convergence. According to Carlson and Herdman (2012), the source of high convergence between measurements is the extent to which the same information is captured, thereby demonstrating high correlation between measuring results. Although the IRAP paradigm and the spatial placement task repeatedly gained the same representational pattern of horizontal spatial metaphors of social status in Chinese, the correlation between the results of the two measurements is not significant, which suggests that even if the two measurements get the same metaphorical pattern, the convergence between them is still not very high.

Liu and Liao (2018) proposed that the embodiment effect has four characteristics—embodied generation level, strength degree, constructing direction, and the online (or offline) task. It is helpful to clarify these four aspects to understand the embodiment effect accurately. The IRAP paradigm and the spatial placement task are different in all aspects except the offline aspects. At a generational level, the IRAP task is involved in the core cognition of nature and culture, which belongs to the macro level, while the core cognition for the spatial placement task is the trunk and limb factor, which belongs to the meso level. The stability is different between the macro and meso levels (Liu and Liao, 2018). For the strength condition, the IRAP paradigm is dominated by rapid automatic processing with more unconscious components, while the spatial placement task has slow and controlled processing with more conscious components. Zestcott et al. (2017) found that consciousness could reduce the embodied effect in social judgment, i.e., reduce the strength of the embodied effect. In the direction of construction, both measurements are unidirectional from the social status of abstract concepts to the horizontal space of concrete concepts. However, what the IRAP paradigm captures is a mental representation at the cognitive level, while the spatial placement task captures the behavioral level, which includes the leftward/rightward executive performance of the embodied behavior variables to the mental representation of social status. In brief, there are differences in the stability, consciousness, and structure of the embodied effect between what the IRAP paradigm captures and what the spatial placement task captures. These differences may lead to distinct spatial metaphorical information captured by the two measurements, which does not result in high convergence. If this inference is correct, perhaps it is beneficial to form new approaches that create new specific experimental paradigms that understand and solve the replication crisis of the embodiment effect (Liu and Liao, 2018).

It is worth noting that the Chinese language mentioned in the study refers to Mandarin. In the future, further checks should be done within different languages and cultures, in order to prove whether the representational pattern of horizontal spatial metaphors for social status gained from this study shows cross-cultural consistency.

## Data Availability Statement

The raw data supporting the conclusions of this article will be made available by the authors, without undue reservation, to any qualified researcher.

## Ethics Statement

Ethical review and approval was not required for the study on human participants in accordance with the local legislation and institutional requirements. Written informed consent for participation was required for this study in accordance with the national legislation and the institutional requirements.

## Author Contributions

HS participated in the design, data analysis, data interpretation, and writing the article. DL participated in the design and data collection. All authors contributed to the article and approved the submitted version.

## Conflict of Interest

The authors declare that the research was conducted in the absence of any commercial or financial relationships that could be construed as a potential conflict of interest.

## Publisher’s Note

All claims expressed in this article are solely those of the authors and do not necessarily represent those of their affiliated organizations, or those of the publisher, the editors and the reviewers. Any product that may be evaluated in this article, or claim that may be made by its manufacturer, is not guaranteed or endorsed by the publisher.
